# Whole Grains, Legumes, and the Subsequent Meal Effect: Implications for Blood Glucose Control and the Role of Fermentation

**DOI:** 10.1155/2012/829238

**Published:** 2011-10-30

**Authors:** Janine A. Higgins

**Affiliations:** Department of Pediatrics, University of Colorado Denver, Anschutz Medical Campus, Aurora, CO 80045, USA

## Abstract

Whole grains and legumes are known to reduce postprandial glycemia and, in some instances, insulinemia. However, the subsequent meal effect of ingesting whole grains and legumes is less well known. That is, inclusion of whole grains or legumes at breakfast decreases postprandial glycemia at lunch and/or dinner on the same day whereas consumption of a whole grain or lentil dinner reduces glycemia at breakfast the following morning. This effect is lost upon milling, processing, and cooking at high temperatures. The subsequent meal effect has important implications for the control of day-long blood glucose, and may be partly responsible for the reduction in diabetes incidence associated with increased whole grain and legume intake. This paper describes the subsequent meal effect and explores the role of acute glycemia, presence of resistant starch, and fermentation of indigestible carbohydrate as the mechanisms responsible for this effect.

## 1. Introduction

Whole grains are those that contain intact cereal germ, endosperm, and bran. Whole grain intake is associated with a variety of beneficial health effects. In large epidemiological studies, whole grain intake is associated with lower body mass index (BMI) [1], and lower incidence of type 2 diabetes, cardiovascular disease [2, 3], and colorectal cancer [4]. Likewise, legume consumption is associated with a reduction in the incidence of type  2 diabetes [5] and, in small prospective intervention studies, with increased glucose tolerance and improved lipemia [6]. One of the mechanisms that may be responsible for the beneficial effects of whole grain and legume consumption is their ability to lower postprandial glucose and insulin responses which, in turn, has effects on hepatic and lipid metabolism [7]. Although the ability of certain whole grains and legumes to lower postprandial glycemia is well documented [8, 9], little attention has been given to the subsequent meal effect of whole grain and legume ingestion. The subsequent or second meal effect is the ability of whole grains and legumes to lower postprandial glycemia not only after the meal at which they are consumed but also at a subsequent meal later in the day or even on the following day. This effect could be useful for blood glucose control in diabetic patients but could also confound insulin dosing regimens by causing an uncalculated or unexpected decrease in insulin requirements at the subsequent meal.

Whole grains and legumes are a collection of different foods with differing structural and physicochemical properties. The amount of insoluble fiber, resistant starch, phytochemicals, granule size, porosity, the interaction of starch and protein within the structural matrix, and other bioactive compounds differs amongst different whole grains and legumes so it is important to examine the effect of different foods on day-long glycemia. Also, given these different properties, it is possible that different whole grains and legumes could possess distinctly different mechanisms of action with regard to the subsequent meal effect. These ideas will be explored in this review, the purpose of which is to describe the subsequent meal effect as it pertains to consumption of whole grains and legumes, discuss the implications for blood glucose control on long-term health, and examine the possible mechanisms whereby whole grains and legumes exert this effect. This is a comprehensive review that utilized all literature examining the subsequent meal effect found using the following search strategy: whole grain plus fermentation or glycemia or insulinemia or (meal and lunch) or (meal and dinner) or (breakfast and lunch) or (dinner and breakfast). If a paper was found that studied any form of whole grain and glycemia at a subsequent meal, it was included. No studies that met this criterion were excluded for any reason.

## 2. Discussion

### 2.1. Subsequent Meal Effect

The subsequent meal effect (or second meal effect) describes the ability of whole grain and legume intake at a single meal to influence postprandial glycemia at the next meal. That is, inclusion of whole grains or legumes at breakfast decreases postprandial glycemia at both lunch and dinner on the same day whereas consumption of a whole grain or lentil dinner reduces glycemia at breakfast the following morning (Table 1). The subsequent meal is always provided as a standardized food or meal with no difference in energy, macronutrient content, or total fiber such that any difference in glycemia following this meal can be attributed to the composition of the first or initial meal.

Barley kernels, rye kernels, and legumes, whether consumed as part of a dinner or breakfast, reduce postprandial glycemia at a subsequent meal (Table 1). Oats and wholemeal bread, which contains processed whole grain material, do not provide a subsequent meal effect (Table 1). Indeed, processing, milling, and cooking at high temperatures may negate the subsequent meal effect or, in some instances, can even exacerbate postprandial glycemia at a subsequent meal. In a study utilizing barley breads cooked under different conditions, only breads containing barley prepared in pumpernickel style, cooked at low temperatures over a long period of time, decreased glycemia at a subsequent meal compared with white wheat bread (WWB) [10]. Bread containing an equivalent amount of barley but cooked under standard bread-baking conditions had no effect on glycemia at a subsequent meal. When barley kernels are milled and cooked in a microwave as porridge, glycemia at the next meal is greater than that observed using WWB as the first meal [11]. Therefore, it is important to consider not only the type of whole grain consumed but also the form of the grain at the time of consumption. Many commercial whole grain products contain highly milled whole grains which have been cooked or extruded to form the final product. Some of these products, such as pasta, result in a structural matrix that decreases postprandial glycemia relative to bread [12]. However, it is possible that some of these milled/cooked foods may not exert a subsequent meal effect on glycemia; so, should initial glycemia play a role in the subsequent meal effect, it seems prudent to advise consumption of whole grains in their native form when possible to elicit the full benefit from their consumption.

All but one study [13] conducted to examine the subsequent meal effect have standardized carbohydrate intake at the first meal by feeding equivalent amounts of available carbohydrate. Generally, 50 g of available carbohydrate was provided per test meal which is equivalent to the amount provided for glycemic index testing. Indeed, the primary goal of many studies was to investigate the subsequent meal effect of meals differing in glycemic index [10, 11, 14, 15, 17, 18]. However, the traditional method of calculation of available carbohydrate is fraught with difficulty, especially in the case of whole grains and legumes which are high in insoluble fiber and resistant starch (RS) [19]. Conventional total fiber assays do not account for all of the RS present in a food such that simple subtraction of total fiber from total carbohydrate, the traditional way of calculating available carbohydrate, may overestimate available carbohydrate in high RS foods. One study did address this issue by assaying both the total fiber and RS content of meals and presenting meals with either 50 g available carbohydrate or 50 g available starch (taking RS into account) [15]. In this study, using barley kernels, adjustment for RS content did not affect the data in any way. However, the RS plus dietary fiber content of the two meals were the same so it is not surprising that there was no difference in effect. It still remains important to consider the method used to calculate available carbohydrate and ensure that calculations are accurate in order to interpret the resultant data.

A confounding factor of the studies conducted thus far is the use of single foods versus complete meals as the initial or first meal. The fat and protein quantity and quality are known to influence the rate of glucose absorption from a mixed meal [20, 21]. Therefore, the dynamics of immediate and subsequent meal glycemia are more complicated if the initial meal is a complete, mixed meal. It seems that serving a single food as the initial meal would simplify data interpretation except for the fact that, under free living conditions, people rarely, if ever, consume a single, stand-alone food as a meal or snack. Because the physiological properties of a food change in relation to other components present in a meal, it is important to examine the effects of whole grain and legume consumption as part of a complete meal. Barely 1/3  of studies conducted thus far utilize complete meals for the initial or first meal (Table 1). Although there is often good agreement between these studies and those utilizing single foods, Wolever et al. [14] showed that lentils fed as a single food decreased glucose area under the curve at a subsequent meal relative to WWB whereas lentils as part of a complete meal did not (Table 1). This data is further convoluted by the fact that the subsequent meals were different: for the lentils as a single food study, the subsequent meal was a drinkable glucose solution whereas the second meal for the lentils as part of a complete meal study was also a complete, mixed meal. Therefore, the form of the subsequent meal could also have influenced the difference in response to lentils in this series of experiments. Clearly, for optimal relevance to the free-living condition and for clarity of data comparison, the initial meal should contain whole grains or legumes as part of a mixed, complete meal and, ideally, the subsequent meal would also be a complete, mixed meal. Further studies are necessary to standardize the optimal conditions for assessing the subsequent meal effect.

### 2.2. Implications for Blood Glucose Control

The subsequent meal effect has important implications for the day-long control of blood glucose and, ultimately, long-term glucose tolerance. The day-long effect of whole grain and legume consumption [11] may be more important to health outcomes than the acute effect at a single meal per se as postprandial hyperglycemia and hyperinsulinemia have been implicated in the development of insulin resistance in both humans and rats. In humans, large postprandial glycemia rises are associated with an increased concentration of free fatty acids in the plasma [22, 23] which causes a decrease in glucose oxidation [22], presumably via the glucose-fatty acid cycle [24], and, ultimately, impairment of insulin sensitivity [25]. Postprandial hyperinsulinemia has also been shown to decrease glucose uptake in muscles and increase glucose uptake in adipose tissue through a change in GLUT 4 mRNA and protein abundance [26, 27] and causes down-regulation of insulin receptors in humans [28]. Thus, attenuation of postprandial glycemia over the course of the day would be expected to have important long-term health implications in healthy adults, particularly with regard to diabetes prevention. 

It is important to note that all studies investigating the subsequent meal effect with whole grains and legumes have used healthy adults as study subjects without exception. In these subjects, glucose tolerance decreased throughout the day [11] which is in contrast to diabetics in whom glucose tolerance increases between morning and evening. Furthermore, diabetic individuals could most benefit from both acute and day-long suppression of glucose peaks as well as the lower rate of increase and decrease in plasma glucose concentrations observed in studies with healthy adults. Therefore, it is important to examine whether the subsequent meal effect of whole grain or legume consumption is apparent in diabetics who are characterized by frank insulin resistance. Although no such studies have been undertaken, a study which examined the effect of meal size on the subsequent meal effect in type 1 diabetic subjects discovered that postprandial glycemia at dinner was higher following a large lunch (50% of daily caloric needs) than a small lunch (25% of daily caloric needs). So, it is reasonable to suspect that the subsequent meal effect of whole grains and legumes may also elicit a measurable effect in diabetic subjects.

If the subsequent meal effect is evident in diabetic individuals, it will be important to determine how this affects insulin dosing throughout the day. It may be necessary to develop an algorithm for the “carb counting” or exchange systems that are commonly employed to estimate insulin doses that incorporates a factor for whole grain or legume consumption at previous meals. Additionally, it may be possible to include whole grains and/or legumes as part of a meal that would normally elicit higher blood glucose concentrations to prevent rises in HbA1c levels. That is, by decreasing glycemia not only following the initial meal but also over the course of the day, the effect of ingesting rapidly absorbed carbohydrates on long-term glycemic control, as measured via HbA1c, may be diminished. Studies investigating this outcome need to be conducted.

It should be noted that while the subsequent meal effect may change insulin dosing requirements in diabetics, it may also be protective against hypoglycemia as the plasma glucose concentration following subsequent meals not only decreases the total glucose response (area under the curve) but also lowers the* rate of decrease* in plasma glucose concentration [14] even in the absence of a difference in glycemia following the initial meal [16]. Also, the plasma glucose concentration prior to the subsequent meal may be slightly higher following whole grain intake than WWB consumption [10, 11] which could prevent hypoglycemia.

### 2.3. Possible Mechanisms

There are many possible mechanisms whereby ingestion of whole grains or legumes could cause lowering of glycemia at a subsequent meal. Some likely candidates are (1) the effect of immediate reductions in glycemia (following the initial meal) on subsequent glucose metabolism/tolerance and, (2) fermentation of indigestible carbohydrate. 

Insoluble fiber present in legumes and whole grains may exert effects on lowering digestion and the rate of absorption of carbohydrates, with consequent lowering of postprandial glycemia, but does not seem to be linked with the subsequent meal effect. In a study which added barley fiber at the same levels found in whole barley kernels to WWB or spaghetti, there was no subsequent meal effect versus WWB [18]. A subsequent meal effect was only observed when twice the amount of barley fiber found in whole barley kernels was added to WWB. Additionally, when whole grain barley was milled into flour and served as porridge, there was a detrimental effect on subsequent meal glycemia despite the presence of the same amount of insoluble and total fiber as in the intact barley kernel which significantly lowered subsequent meal glycemia [11]. Thus, it is unlikely that insoluble fiber *per se* plays a significant role in the mechanism responsible for the subsequent meal effect. 

#### 2.3.1. Immediate Reductions in Glycemia Following the Initial Meal

In many cases, postprandial glycemia following the initial meal, or immediate glycemia, is lowered by whole grain and legume intake and influences subsequent meal glycemia (e.g., [10, 11, 14]). In these studies there is a direct correlation between the reduction in immediate glycemia and the magnitude of the second meal effect. So, under these conditions, immediate glycemia seems to play a role in facilitating the second meal effect. It has been hypothesized that lower postprandial glycemia decreases oxidative stress by attenuating cytokine production in healthy adults [11] which has been shown to impair insulin signaling [29]. The effect of glycemia on circulating free fatty acid concentrations, which are associated with impaired insulin action and, therefore, lower glucose sensitivity [30], may also contribute to the subsequent meal effect. In one study, whole grain consumption at dinner significantly decreased fasting free fatty acid concentrations [17] whereas another found that, in response to a lentil dinner, there was no difference in fasting free fatty acid concentrations the following morning yet still there was a second meal effect [14]. Evidently, this is a possible mechanism of action but there is a paucity of data to support this theory, and further studies need to be conducted to determine how significant this mechanism might be in eliciting the subsequent meal effect. 

It is reported in the literature that immediate glycemia is an important mechanism for the second meal effect when the test meal is breakfast and the subsequent meal is lunch. That is, when the two meals are only several hours apart. However, in studies where subsequent meal glycemia is closely linked with initial meal glycemia, two are breakfast-lunch studies [10, 11] whereas one is a dinner-breakfast study with a much longer time between the test meals [14]. Additionally, in the overnight condition, the subsequent meal effect can occur in the absence of any immediate change in glycemia following the initial meal and the subsequent meal effect under these conditions may stem from carbohydrate fermentation in the large bowel. 

A decrease in immediate glycemia following the initial meal is not obligatory for the subsequent meal effect to occur, even in a breakfast-lunch paradigm [16] and, conversely, a decrease following an initial breakfast meal does not equate to a reduction in glycemia at the subsequent meal [13]. In this study [13], a lentil breakfast significantly reduced postprandial glycemia following a standard lunch but WWB nibbled over time to mimic the glycemia observed upon lentil ingestion did not result in a subsequent meal effect. Thus, although there are plausible mechanisms by which reduced immediate glycemia could contribute to both the subsequent meal effect and the reduction in diabetes and cardiovascular disease incidence observed with high whole grain intake, the direct evidence for immediate glycemia as a mechanism for the subsequent meal effect is, at best, equivocal.

#### 2.3.2. Fermentation of Indigestible Carbohydrates

Fermentation of indigestible carbohydrates produces SCFA which have been associated with improved insulin sensitivity and glucose tolerance due to decreased hepatic glucose output and free fatty acid concentrations [31]. In studies examining the role of whole grain and legume consumption on the subsequent meal effect, those that measure fermentation find a strong association between fermentation and reduced glycemia at a subsequent meal. In fact, in all cases, a reduction in glycemia at a subsequent meal is associated with a significant increase in carbohydrate fermentation when measured (Table 1). Also, although fermentation has been rejected as a mechanism for the subsequent meal effect in breakfast-lunch studies with a protracted time-frame, there have been reports of measurable fermentation at the lunch meal, particularly in the case of rye kernel ingestion at breakfast [11]. In a more thorough investigation of this concept, it was found that significant concentrations of acetate appear in the blood within 4 hours of barley kernel ingestion whereas butyrate and propionate appear later (6–15 h after ingestion) [32]. Thus, it is likely that the acetate fraction of SCFA could be partly responsible for the subsequent meal effect in breakfast-lunch studies whereas butyrate and propionate may be more important for modulating carbohydrate tolerance over a longer time frame in dinner-breakfast studies. 

In support of fermentation as the most significant contributor to the subsequent meal effect are numerous studies finding that the different particle sizes and composition of whole grains produce different amounts of SCFA with smaller, processed samples not causing an increase in butyrate concentrations [33]. These data are consistent with the finding that intact whole grains elicit the strongest subsequent meal effect which is lost upon extensive milling. It is important to note that, in mixed meal studies, other food components such as protein can modulate or facilitate fermentation and the interaction between food components may also, therefore, contribute to the subsequent meal effect through their effects on fermentation. 

A single study has directly investigated the effect of fermentation on subsequent meal glycemia [34]. The test meals in this study contained either nonfermentable amylopectin plus cellulose, amylopectin plus fermentable lactulose, or fermentable amylose plus cellulose. Meals containing these ingredients were presented at breakfast with subsequent meal glycemia measured following a standardized lunch meal five hours later. Both of the meals containing fermentable carbohydrate decreased subsequent meal glycemia relative to the amylopectin meal [34]. The amylose meal also decreased immediate glycemia and insulinemia (following the first meal, breakfast) whereas the lactulose meal did not. The lactulose meal, however, caused a significant increase in nonesterified fatty acid concentrations and gastric emptying time following the initial meal which the amylose meal did not [34]. Therefore, the mechanism for the subsequent meal effect involved fermentation and decreased initial glycemia/insulinemia for the amylose meal whereas fermentation plus changes in gastric emptying rate and non-esterified fatty acid concentrations seem to cause this effect for the lactulose meal. Thus, it seems that many factors may be involved in eliciting the subsequent meal effect with fermentation of indigestible carbohydrate acting as a common, key modulator.

### 2.4. The Resistant Starch Caveat

RS has the intrinsic properties of both soluble and insoluble fiber. As such, it can decrease transit time through the gut and increase stool bulk but is also an excellent substrate for fermentation in the large bowel which decreases bowel pH and generates short chain fatty acids (SCFA). RS is known to decrease plasma glycemia and insulinemia following ingestion [35]. Therefore, the RS content of whole grains and legumes may facilitate the second meal effect, primarily through fermentation in the bowel or due its effect on postprandial glycemia and insulinemia. Likely, both mechanisms are involved during RS consumption. Test meals high in RS have elicited a strong subsequent meal effect both during a breakfast-lunch paradigm, where postprandial may play a role in facilitating this effect [11], and a dinner-breakfast model, in which fermentation seems to be the predominate driving factor [18]. Although there is scant evidence to support the role of immediate postprandial glycemia reduction as a mechanism for the subsequent meal effect, breakfast-lunch studies where the time between meals is 4 h, are probably too short to assess the effects of RS fermentation which starts at about 6–8 h following ingestion in healthy adults [36]. Therefore, factors beyond fermentation may play a role in eliciting the subsequent meal effect in response to high RS foods. 

Most studies examining the subsequent meal effect of whole grain or legume intake do not independently report the RS content of the test meals. Rather, a combined RS plus dietary fiber number is provided. In those studies that do report RS as a discrete variable in the diet [10, 11, 18], only one has adequate controls to determine if RS exerts any independent effect beyond total fiber or RS + total fiber [11]. By comparing the data in this study from barley kernels (8 g RS, 9.1 g, total fiber, 17.1 g RS + total fiber), rye kernels (matched to barley for RS + total fiber but lower in RS; 3.7 g RS, 14.2 g total fiber, 17.9 g RS + total fiber), and barley porridge (matched to barley for total fiber but low in RS; 1.7 g RS, 9.2 g total fiber, 10.9 g RS + total fiber), it can be seen that the RS content of whole grains may be an independent contributor to the subsequent meal effect. Only the barley kernel breakfast significantly increased prelunch basal glucose concentration relative to WWB and, while both barley and rye kernel meals decreased total day-long glucose area under the curve, barley kernels had a larger effect. Finally, barley porridge which is low in RS but matched to barley kernels for total fiber produced no subsequent meal effect [11], indicating that RS exerts an effect beyond that of fiber.

## 3. Conclusions

The ingestion of whole grains and legumes may cause diminution of postprandial glycemia not only at the meal in which they were consumed but also at subsequent meals. This effect is apparent whether whole grains and legumes are consumed during breakfast, causing decreased glycemia for the remainder of the day, or at dinner, causing lower glycemia at breakfast the following morning. This effect may prove useful in public health efforts for diabetes prevention and could be a factor in the documented relationship between whole grain intake and lower risk of diabetes. Additionally, by decreasing glycemia not only following the initial meal but also over the course of the day, the effect of ingesting rapidly absorbed carbohydrates on long-term glycemic control, as measured via HbA1c, may be diminished if whole grains or legumes are also consumed. This is a hypothesis that warrants further investigation. It is important to note that extensive milling or cooking negates the subsequent meal effect so whole grains and legumes should be consumed in their native state or with minimal processing for full benefit. Finally, it may be concluded that fermentation of indigestible carbohydrate is the primary mechanism whereby whole grains and legumes exert the subsequent meal effect.

## Figures and Tables

**Table 1:** Subsequent meal effect of whole grains and lentils.

Study	Initial/ 1st meal	Subsequent meal	Time between meals (h)	Control food^#^	Test food	Complete meal (Y/N)	Effect of test food on glucose at subsequent meal	Breath hydrogen (fermentation)
Jenkins et al.* [13]	B/F	Lunch	4	WWB	Lentils	Y	↓ AUC by 38%	↑ 200%

Wolever et al. [14]								
(1)	Dinner	B/F	?	Glucose	Lentils	N	↓ AUC	N/A
(2)	Dinner	B/F	?	WWB	Whole meal bread	N	=	
(3)	Dinner	B/F	?	Bread and potato	Lentil and barley	Y	= AUC; ↓ mean postprandial [G]	

Liljeberg et al. [10]	B/F	Lunch	4	WWB	Barley bread (long, slow cooking) + BF	Y	↓ only with added BF, not barley bread alone	N/A

Granfeldt et al. [15]	Dinner	B/F	?	WWB	Barley kernels	N	↓ AUC	N/A

Samra and Anderson [16]	B/F	Lunch^†^	1.25	WWB/ Cornflakes	Fiber One cereal	Y	↓ AUC	

Nilsson et al. [17]	Dinner	B/F	10.5	WWB	Barley kernels orcut barley	N	↓ AUC by 28%	↑
							↓ peak [G]	

Nilsson et al. [18]	Dinner	B/F	10.5	WWB/ Spaghetti	Spaghetti + high-dose BF	N	↓ AUC	↑

Nilsson et al. [11]	B/F	Lunch	4	WWB	Rye kernels	N	↓ AUC	↑
					Oat		= AUC	=
					Barley kernels		↓ AUC	↑
		Dinner	10.5	WWB	Rye kernels	N	↓ AUC	=
					Oat		= AUC	↑
					Barley kernels		↓ AUC	↑

B/F: breakfast; AUC: area under the curve; WWB; white wheat bread; BF: barley fiber; [G]: glucose concentration.

All meals matched for available CHO except for*.

^#^Control foods were not prepared with whole grains.

^†^The timing of the subsequent meal (1.25 h after BF) is too short for the second meal to be considered “lunch”.
